# Cancer Care During COVID-19 Era: The Quality of Life of Patients With Thyroid Malignancies

**DOI:** 10.3389/fonc.2020.01128

**Published:** 2020-06-23

**Authors:** Rosa Falcone, Giorgio Grani, Valeria Ramundo, Rossella Melcarne, Laura Giacomelli, Sebastiano Filetti, Cosimo Durante

**Affiliations:** ^1^Department of Translational and Precision Medicine, Sapienza University of Rome, Rome, Italy; ^2^Department of Surgical Sciences, Sapienza University of Rome, Rome, Italy

**Keywords:** COVID-19, cancer care, quality of life, emotional outbreak, outcomes

## Abstract

**Background:** The Covid-19 pandemic's potential psychological impact has been widely discussed on the basis of expert opinion and previous experience with emergencies of this type. We conducted a survey of cancer patients to explore more objectively the outbreak's impact on their emotional well-being and quality of life.

**Methods:** Between March 18 and April 4, 2020, at an endocrine cancer center in Rome, Italy, 137 patients were asked to complete an online 6-item questionnaire developed by our staff to explore the emotional effects of the Covid-19 outbreak in Italy (Covid-19 Emotional Impact Survey, C-19EIS). For validation purposes, we also asked participants to complete an online version of the validated Italian translation of the EORTC QLQ-C30 questionnaire. Responses were analyzed in relation to responders' age, sex, and clinical status (advanced/metastatic disease undergoing systemic treatment vs. stable metastatic thyroid cancer in active surveillance vs. low-risk thyroid cancers with no evidence of structural disease during standard follow-up).

**Results:** Response rates were high (51% for the C-19EIS, 44.5% for the EORTC QLQ-C30). Overall C-19EIS scores indicated high concern over the outbreak (median 8/12). Scores were higher in women (8 [IQR 5–9] vs. 6 [IQR 5–8] in men; *p* = 0.048) and in patients <65 years (8 [IQR 5–9] vs. 6 [IQR 4–8] in older patients; *p* = 0.013). No differences emerged across clinical status groups. C-19EIS scores were inversely correlated with the EORTC QLQ-C30 Emotional function subscale (rho −0.69; *p* < 0.001).

**Conclusions:** There is objective evidence that the Covid-19 outbreak is causing substantial emotional distress among cancer patients, regardless of their disease severity or current health-care needs.

## Introduction

The severe acute respiratory syndrome coronavirus 2 (SARS-CoV-2) and the disease it causes, Coronavirus disease 2019 (Covid-19), have generated a public health emergency of global proportions (1). In Italy, one of the countries hit hardest by the pandemic, the number of confirmed cases of Covid-19 rose sharply in early March 2020, reaching 162,004 at the time of this writing, with a death rate of 12.7% (2). On March 8, 2020, the Italian government began implementing extraordinary measures aimed at slowing the spread of the virus. All but the most essential public services and commercial activities were abruptly suspended. “Social distancing norms” became the new by-word for asymptomatic individuals (3). To avoid further spread of the disease among staff members and hospital inpatients, physicians were forced to limit non-urgent procedures and encounters with patients (4–8).

Our unit is dedicated to the treatment and follow-up of individuals diagnosed with cancer (mainly endocrine tumors). In early March, we began reviewing upcoming encounters scheduled with our patients and planning alternative forms of contact and consultation based on phone calls, emails, and video calls. Service would be guaranteed for patients requiring immediate care (e.g., a new cycle of systemic anticancer drug therapy). For others, visits or procedures would be rescheduled as soon as possible or canceled until further notice, depending on the urgency of their needs.

From the outset, we were acutely aware of the risk that these changes (and the Covid-19 outbreak itself) could have moderate-to-severe adverse effects on our patients' psychological well-being (9). SARS-CoV-2 infections in patients with cancer appear more likely to cause severe morbidity and mortality, owing in part to these individuals' more advanced age and in part to the immunosuppressive effects of their malignancies and the drugs, radiation, and surgical procedures used to treat them (10–12). Indeed, active anticancer treatment is one of the risk factors for severe illness from Covid-19 cited by the U.S. Centers for Disease Control and Prevention (13).

Information of this type penetrates our patients' homes daily and in various forms. The constant inflow of “facts” and “numbers”—potentially contradictory, frequently lacking adequate context or explanation by experts—can generate confusion, increased anxiety, heightened stress responses, and downstream effects on health (14). In Italy, access to hospital care for cancer patients is guaranteed, but the patients themselves remain torn between the fear of hospital exposure to SARS-CoV-2 and the fear of potentially untreated progression of their disease if their visits are delayed or canceled.

Several publications have highlighted the likelihood that the Covid-19 pandemic will have negative short-to-medium-term emotional effects in a variety of vulnerable subgroups. Thus far, however, hard data documenting this outcome in affected populations are lacking. This study was undertaken to explore more objectively the effects of this potentially life-threatening pandemic on cancer patients' perceptions and feelings.

## Methods

During the first week of March 2020, patients being followed by our staff were contacted by telephone and notified of any changes that would be made in their appointments for the coming months, as a result of the Covid-19 outbreak. For patients with email addresses who had been personally affected by the scheduling changes, we also sent links to two online questionnaires regarding the quality of their lives since the beginning of the pandemic, with a personal invitation from staff clinicians to complete both surveys. The first link opened the Covid-19 Patient Impact Survey, a 21-item questionnaire designed *ad hoc* by our team to explore and measure the emotional/overall impact of the rapidly escalating Covid-19 epidemic in Italy. The second link allowed access to the validated Italian translation of the European Organization for Research and Treatment of Cancer Quality of Life Questionnaire (EORTC QLQ-C30) (15), which most patients had already completed during a previous encounter with our staff. Both online questionnaires were accompanied by informed consent forms which had to be signed by the patient and submitted with their responses. Clinical data were collected as part of a prospective, observational study on the outcome of thyroid cancer patients (NCT04031339). The academic use of the EORTC questionnaire was authorized by the Quality of Life Group (ID 67335). The study was conducted in a thyroid cancer center at the Department of Translational and Precision Medicine of Sapienza University of Rome, Italy. Between March 18 and April 4, 2020, non-responders were re-contacted by phone by the clinicians themselves, who reviewed the scheduling changes being implemented with the patient and renewed the invitation to take part in the survey.

For the purposes of our analysis, patients were grouped as follows, on the basis on their clinical status and the manner in which they were being followed by our staff [The protocols routinely used in our unit are based on currently recommended practices (16) and have been described elsewhere (17, 18)]. Group 1 included patients with progressive metastatic cancers that were being treated with systemic anticancer drugs. These individuals normally come into the clinic monthly for a clinical evaluation, and all were thus affected by our revised appointment schedules. Group 2 patients had apparently stable metastatic thyroid cancer, which was being managed with active surveillance and required no immediate therapeutic intervention (e.g., non-progressive or low-burden disease). Patients in Group 3 had no evidence of structural disease after initial treatment or small, local, non-threatening (19) papillary thyroid cancers that were being managed non-surgically (i.e., with active surveillance alone). Both types of patient were generally seen by our staff once a year.

### Scale Calculations and Statistical Analysis

The 21-item Covid-19 Patient Impact Survey included a 6-item Core Component (Covid-19 Emotional Impact Survey, C-19EIS) designed to explore and quantify the outbreak's emotional impact on our cancer patients. Responses to all six questions were mandatory for participation in the survey. For each of the four yes/no questions, we assigned one point for a positive answer and zero points for a negative response. For the two multiple-choice questions, the number of points assigned to each answer ranged from 0 to 4. The points assigned for the responses to these six items were then summed to produce a Covid-19 Concern Score, which ranged from 0 (not at all concerned) to 12 (very much concerned). For some questions, additional space was provided for optional comments or details volunteered by the responder. Differences in responses to individual items related to age, sex, or clinical status were also assessed with a chi square test or Fisher exact test, as appropriate.

In addition to the Core Component responses, we also analyzed responses to the final question of the Covid-19 Patient Impact Survey itself, regarding patient satisfaction with the care they were receiving at the time of the survey.

The EORTC QLQ-C30 consists of 30 items representing a global health/quality of life (QoL) subscale, five function subscales (physical, role, emotional, cognitive, and social), and nine symptom subscales/items (The latter subscales were not considered in our analyses, owing to the specific composition of our cohort). We calculated the subscale scores (range: from 0 to 100) for our responders and dealt with missing data in accordance with instructions in the EORTC QLQ-C30 scoring manual (20). The results were compared with published normative data (21). For the subset of Group 1 patients who had already responded to the EORTC QLQ-C30 on a previous occasion, we also evaluated score changes over time using the related-sample Wilcoxon signed rank test. Non-parametric tests were used due to the small sample size.

The Spearman rank correlation coefficient technique was used to explore the correlation between patient responses to the C-19EIS and the EORTC QLQ-C30 subscales. Between-group differences in the Covid-19 Concern scores and in the QLQ-C30 subscale scores were evaluated using the Kruskal-Wallis test (differences related to clinical status) or the Mann-Whitney U test (age- or sex-related differences). All data were analyzed with IBM SPSS Statistics program, version 25.0 (IBM Corp., Armonk, NY, US).

## Results

E-mail invitations to take part in our survey were sent to a total of 137 cancer patients. Seventy of these individuals returned completed the C-19EIS, and 61 also completed the EORTC QLQ-30 (response rates 51 and 44.5%, respectively). Table 1 summarizes the characteristics of the 70 responders and those of responder subgroups defined by age, sex, or clinical status. The significant over-representation of males among the patients in Group 1 (68%; *p* < 0.001) is consistent with the well-documented predominance of this sex among cancer patients with the most severe/aggressive forms of disease.

**Table 1:** Clinical features of the study cohort.

		**All patients**	**Age**			**Sex**			**Clinical Status**			
			**<65 years**	**≥65 years**	***p***	**Female**	**Male**	***p***	**Group 1**	**Group 2**	**Group 3**	***p***
	**n**	**70**	**45**	**25**		**40**	**30**		**25**	**21**	**24**	
Sex	Males	30	18	12	0.62	–	30	–	17	10	3	**<0.001**
		42.9%	40.0%	48.0%		–	100.0%		68.0%	47.6%	12.5%	
	Females	40	27	13		40	–		8	11	21	
		57.1%	60.0%	52.0%		100.0%	–		32.0%	52.4%	87.5%	
Age	Years (IQR)	57 (48–69.75)	51 (45.5–57)	75 (69–79)	<0.001	57 (48–65.75)	59 (48.5–73.25)	0.51	61 (50.5 – 72.5)	59 (46.5 – 72)	56 (48.25 – 68)	0.53
Months from diagnosis	Median (IQR)	107.5 (39.7–164.5)	91 (40.5–162.5)	120 (38.5–174.5)	0.64	123.5 (46.25–188)	78 (35.75–128.25)	0.09	77 (33–129.5)	123 (31–188)	116.5 (58.25–167.25)	0.23
Previous treatments	Surgery	68	44	24	1.00	39	29	1.00	23	21	24	0.16
		97.1%	97.8%	96.0%		97.5%	96.7%		92.0%	100.0%	100.0%	
	Radioiodine	41	24	17	0.31	24	17	0.78	13	13	15	0.71
		58.6%	53.3%	68.0%		60.0%	56.7%		52.0%	61.9%	62.5%	
	Anticancer drugs	25	14	11	0.31	8	17	**0.002**	25	0	0	**<0.001**
		35.7%	31.1%	44.0%		20.0%	56.7%		100.0%	0.0%	0.0%	
Diagnosis^*^	Papillary thyroid cancer	40	27	13	0.24	29	11	**0.04**	7	9	24	**<0.001**
		57.1%	60.0%	52.0%		72.5%	36.7%		28.0%	42.9%	100.0%	
	Follicular thyroid cancer	5	2	3		1	4		2	3	0	
		7.1%	4.4%	12.0%		2.5%	13.3%		8.0%	14.3%	0.0%	
	Poorly-differentiated thyroid cancer	5	2	3		1	4		5	0	0	
		7.1%	4.4%	12.0%		2.5%	13.3%		20.0%	0.0%	0.0%	
	Medullary thyroid cancer	15	12	3		6	9		6	9	0	
		21.4%	26.7%	12.0%		15.0%	30.0%		24.0%	42.9%	0.0%	
	Anaplastic thyroid cancer	1	0	1		0	1		1	0	0	
		1.4%	0.0%	4.0%		0.0%	3.3%		4.0%	0.0%	0.0%	
	Adrenal cancer	3	2	1		2	1		3	0	0	
		4.3%	4.4%	4.0%		5.0%	3.3%		12.0%	0.0%	0.0%	
	NSCLC	1	0	1		1	0		1	0	0	
		1.4%	0.0%	4.0%		2.5%	0.0%		4.0%	0.0%	0.0%	
Stage of cancer at survey	I	20	15	5	0.29	17	3	**<0.001**	0	0	20	**<0.001**
		28.6%	33.3%	20.0%		42.5%	10.0%		0.0%	0.0%	83.3%	
	II	6	3	3		6	0		2	0	4	
		8.6%	6.7%	12.0%		15.0%	0.0%		8.0%	0.0%	16.7%	
	III	3	3	0		2	1		0	3	0	
		4.3%	6.7%	0.0%		5.0%	3.3%		0.0%	14.3%	0.0%	
	IV	41	24	17		15	26		23	18	0	
		58.6%	53.3%	68.0%		37.5%	86.7%		92.0%	85.7%	0.0%	

*NSCLC, non-small-cell lung cancer; IQR, interquartile range*.

* *The patients followed by our staff have thyroid cancers or other endocrine cancers. The patient listed here with a diagnosis of NSCLC is also being followed in our unit because, like several of our endocrine cancer patients, she is enrolled in a basket trial for tumors harboring RET mutations (She is also suffering from a paraneoplastic syndrome). The bold values referer to statistically significant variables*.

### Covid-19 Patient Impact Survey

**Figure 1:**
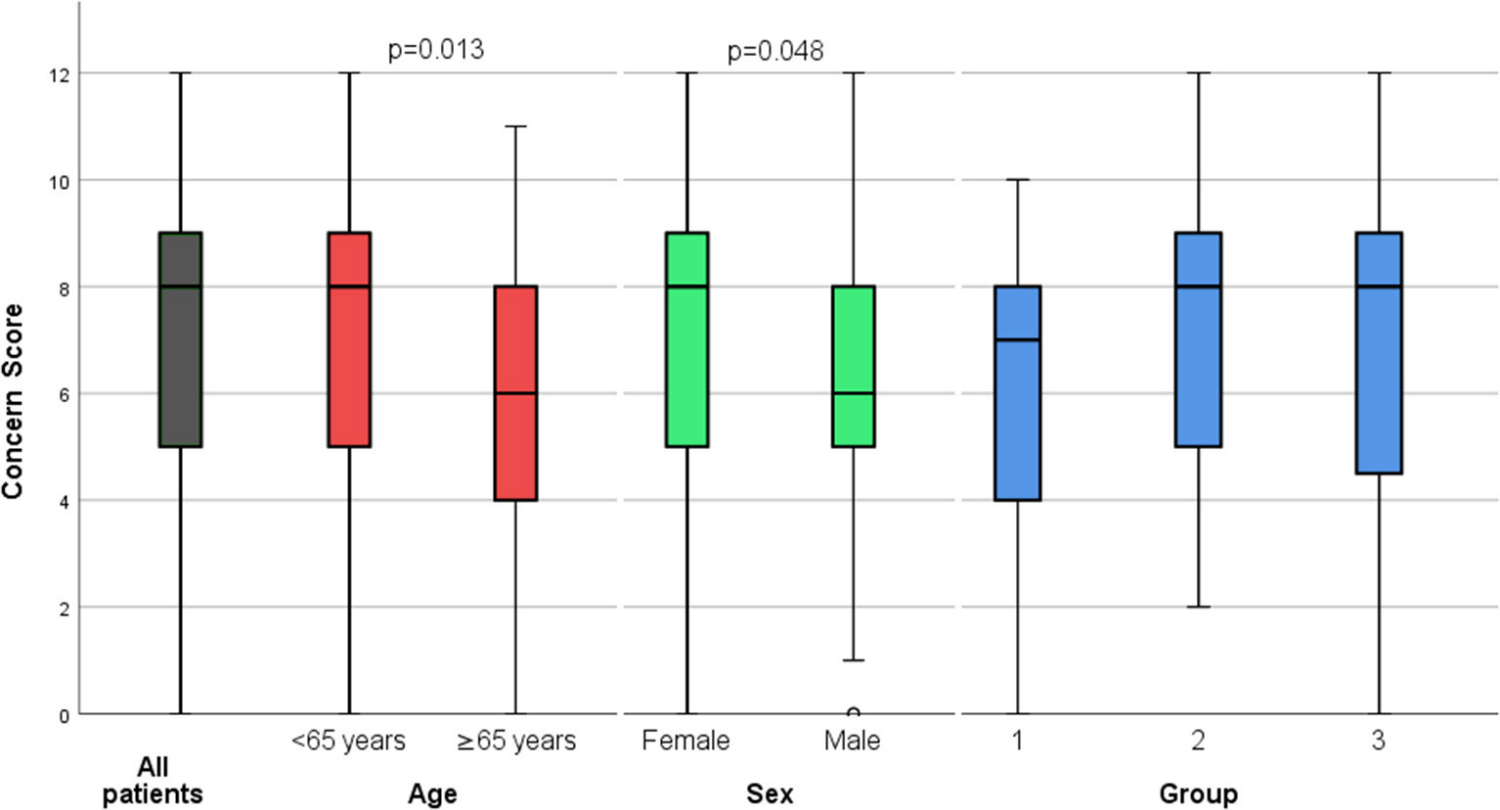
Box-and-whisker plots showing Covid-19 Concern Scores. Results (median, IQR) are shown for the study cohort as a whole (gray bar) and for subgroups defined by age (red bars), sex (green bars), and clinical status (blue bars).

Responses to each of the six questions in the C-19EIS are summarized in Table 2. The median Covid-19 Concern score was 8/12 (IQR 5–9). Scores were significantly higher in women (median: 8 [IQR 5–9] vs. 6 [IQR 5–8] in men; *p* = 0.048) and in patients under 65 years of age (median 8 [IQR 5–9] vs. 6 [IQR 4–8] in those aged 65 or over; *p* = 0.013). No significant score differences were observed across clinical status groups (Figure 1). Analysis of answers to single questions also revealed certain sex-related and age-related differences. Compared with the men, women were more likely to report fear (77.5% vs. 50%; *p* = 0.02) and/or substantial emotional stress (“quite a bit” or “very much” 67.5% vs. 26.7%; *p* = 0.008) as a result of the outbreak (Table 2).

**Table 2:** Answers to the core emotional component of the Covid-19 patient impact survey.

		**All patients**	**Age**			**Sex**			**Clinical Status**			
			**<65 years**	**≥65 years**	***p***	**Female**	**Male**	***p***	**Group 1**	**Group 2**	**Group 3**	***p***
	**n**	**70**	**45**	**25**		**40**	**30**		**25**	**21**	**24**	
**Covid-19 Core Emotional Component**												
1. Are you experiencing fear / anxiety related to the Covid-19 pandemic?	No	24	13	11	0.29	9	15	**0.02**	12	6	6	0.19
		34.30%	28.9%	44.0%		22.5%	50.0%		48.00%	28.57%	25.00%	
	Yes	46	32	14		31	15		13	15	18	
		65.70%	71.1%	56.0%		77.5%	50.0%		52.00%	71.43%	75.00%	
2. Has the onset of the Covid-19 outbreak left you feeling less medically protected?	No	31	20	11	1.00	17	14	0.81	11	11	9	0.60
		44.30%	44.4%	44.0%		42.5%	46.7%		44.00%	52.38%	37.50%	
	Yes	39	25	14		23	16		14	10	15	
		55.70%	55.6%	56.0%		57.5%	53.3%		56.00%	47.62%	62.50%	
3. Do you believe your disease will be affected by the Covid-19 outbreak?	No	41	26	15	1.00	26	15	0.23	15	11	15	0.78
		58.60%	57.8%	60.0%		65.0%	50.0%		60.00%	52.38%	62.50%	
	Yes	29	19	10		14	15		10	10	9	
		41.40%	42.2%	40.0%		35.0%	50.0%		40.00%	47.62%	37.50%	
4. Has the Covid-19 outbreak changed how you perceive your disease?	No	47	28	19	0.29	29	18	0.44	16	15	16	0.87
		67.10%	62.2%	76.0%		72.5%	60.0%		64.00%	71.43%	66.67%	
	Yes	23	17	6		11	12		9	6	8	
		32.90%	37.8%	24.0%		27.5%	40.0%		36.00%	28.57%	33.33%	
5. How much impact is the Covid-19 outbreak having on the quality of your life?	(0) Little or none	10	4	6	0.07	5	5	0.08	6	1	3	0.42
		14.30%	8.9%	24.0%		12.5%	16.7%		24.00%	4.76%	12.50%	
	(2) Some	24	13	11		10	14		8	10	6	
		34.30%	28.9%	44.0%		25.0%	46.7%		32.00%	47.62%	25.00%	
	(3) Quite a bit	27	20	7		17	10		9	7	11	
		38.60%	44.4%	28.0%		42.5%	33.3%		36.00%	33.33%	45.83%	
	(4) Very much	9	8	1		8	1		2	3	4	
		12.90%	17.8%	4.0%		20.0%	3.3%		8.00%	14.29%	16.67%	
6. How much impact is the Covid-19 outbreak having on your emotional state?	(0) Little or none	11	4	7	0.10	5	6	**0.008**	6	1	4	0.30
		15.70%	8.9%	28.0%		12.5%	20.0%		24.00%	4.76%	16.67%	
	(2) Some	24	15	9		8	16		10	8	6	
		34.30%	33.3%	36.0%		20.0%	53.3%		40.00%	38.10%	25.00%	
	(3) Quite a bit	22	15	7		17	5		6	9	7	
		31.40%	33.3%	28.0%		42.5%	16.7%		24.00%	42.86%	29.17%	
	(4) Very much	13	11	2		10	3		3	3	7	
		18.60%	24.4%	8.0%		25.0%	10.0%		12.00%	14.29%	29.17%	
**Other question**												
Do you feel the hospital staff could do more to support you during this emergency?	Yes	7	7	0	**0.04**	6	1	0.23	0	3	4	0.11
		10.00%	15.6%	0.0%		15.0%	3.3%		0.00%	14.29%	16.67%	
	No	63	38	25		34	29		25	18	20	
		90.00%	84.4%	100.0%		85.0%	96.7%		100.00%	85.71%	83.33%	

*The bold values referer to statistically significant variables*.

At the time of the survey, most responders reported being satisfied with the support they had received from health-care professionals since the beginning of the pandemic (Table 2). This response was significantly more common among the older participants (100% vs. 84.4% of the younger patients; *p* = 0.04).

Including four covariates (the clinical group, current stage, age, and gender) in a multivariate linear regression analysis, only age resulted to be a significant predictor of C-19EIS (Table 3). Since clinical group and stage are strongly correlated, the analysis was repeated including only one of these two variables a time. Furthermore, the same model was revised to include only a measure of social support (living with a partner or family member; Table 4). However, only age was retained as a significant predictor.

**Table 3:** Multivariate linear regression analysis: predictors of the C-19EIS score.

	**Beta Coefficient**	***p***
Sex	0.14 (−0.77 to 2.51)	0.30
Age	**−0.27 (−0.11 to** **−0.08)**	**0.02**
Stage	0.09 (−0.75 to 1.15)	0.67
Group	0.15 (– 0.97to 2.06)	0.47

*The bold values referer to statistically significant variables*.

**Table 4:** Multivariate linear regression analysis, including social support (living situation): predictors of the the C-19EIS score.

	**Beta Coefficient**	***p***
Sex	0.20 (−0.45 to 2.91)	0.15
Age	– **0.24 (**– **0.001 to** –**0.10)**	**0.05**
Stage	0.13 (– 0.64 to 1.24)	0.52
Group	0.19 (– 0.83 to 2.18)	0.37
Living situation	−0.20 (−4.02 to 0.38)	0.10

*The bold values referer to statistically significant variables*.

### EORTC Quality of Life Questionnaire—C30

Supplementary Figure 1 summarizes the results of the 61 completed EORTC QLQ-C30. Mean global health status/QoL scores and function subscale scores for our responders were similar to those of individuals represented by the European general population norms (21). However, the physical and role function scores for Group 1 patients were significantly lower than the general population norms. The Group 1 scores were also significantly lower than those for Groups 2 and 3 (physical function: 73.3 [IQR 60–91.7] vs. 93.3 [IQR 70–100] and 86.7 [IQR 81.7–98.3], respectively; *p* = 0.01, and role function: 75 [IQR 50–100] vs. 83.3 [IQR 83.3–100] and 100 [IQR 83.3–100], respectively; *p* = 0.03).

For the 18 patients of Group 1 who had completed an EORTC QLQ-C30 questionnaire prior to December 2019 (onset of Covid-19 outbreak in China), scores from the present survey revealed no significant changes over time in the patients' global health/QoL or functional statuses (Table 5).

**Table 5:** Differences in the Global health/Quality of Life scale of the EORTC QLQ-C30 instrument and in Function subscales, longitudinally evaluated in Group 1 patients.

	**Previous evaluation**	**Current evaluation**	***p***
Global health status/QoL	66.7 (50 to 83.3)	75 (47.9 to 83.3)	0.53
Physical Function	80 (53.3 to 93.3)	73.3 (60 to 93.3)	0.55
Role Function	91.7 (58.3 to 100)	83.3 (62.5 to 100)	0.57
Emotional Function	87.5 (50 to 100)	83.3 (54.2 to 91.7)	0.21
Cognitive Function	100 (83.3 to 100)	100 (83.3 to 100)	0.56
Social Function	100 (66.7 to 100)	100 (66.7 to 100)	0.83

### Covid-19 Emotional Impact Survey Validation

Data supporting the reliability of our C-19EIS data are summarized in Table 6. The Covid-19 Concern Score displayed a strong inverse correlation with the Emotional Function subscale of the EORTC (rho −0.69; *p* < 0.001), a moderate inverse correlation with the EORTC QLQ-C30 Global health/QoL scale score (rho −0.40; *p* = 0.001), and a weak inverse correlation with the Social Function subscale (rho −0.37, *p* = 0.003).

**Table 6:** Correlations (Spearman's rho) between the COVID-19 Concern score, the Global health/Quality of Life scale of the EORTC QLQ-C30 instrument and its Function subscales.

**Correlation with Concern Score**	**Spearman's rho**	***p***
Global health status/QoL	−0.40	0.001
Physical Function	0.09	0.49
Role Function	−0.23	0.07
Emotional Function	−0.69	<0.001
Cognitive Function	0.10	0.42
Social Function	−0.37	0.003

## Discussion

Previous studies have revealed a wide range of psychosocial impacts on people at the individual, family and community levels during the outbreak of a viral epidemic (9, 22). Increasing attention is being focused on the psychological effects of the Covid-19 pandemic on the general population and specific subgroups, including frontline healthcare providers (23), children, university students, and older adults with psychiatric conditions (9, 24). Thus far, however, no data have been reported on this issue in cancer patients, a group already subjected to substantial emotional stress related to their disease and widely recognized to be at increased risk for contracting and dying from Covid-19 (25). To the best of our knowledge, ours is also the first attempt to address this knowledge gap and to document the results based on reports by the patients themselves. Almost all the patients we surveyed had thyroid cancers, which are generally indolent tumors associated with a good-to-excellent prognosis (26, 27). Our cohort provides a window onto the emotional states of patients with prognostically diverse forms of cancer, ranging from those with advanced, progressive disease associated with clearly reduced chances of survival all the way down to those who appear to be “cured” and whose likelihood of survival is similar to that of the general population.

Interestingly, the emotional distress provoked by the Covid-19 outbreak was strikingly similar across the entire broad range of disease statuses represented in our cohort, with Concern Scores ranging from 7 to 8 out of 12. In the multivariate analysis, disease severity and stage were not significant predictors of C-19EIS. Indeed, slightly (but not significantly) lower Covid-19 concern scores were seen in the Group 1 patients—those with the most advanced disease, the most concrete medical needs, and the most obviously reduced physical and role functions. Furthermore, analysis of the subset of Group 1 patients with previous EORTC QLQ-C30 data revealed that their overall QoL had been relatively unaffected by the added stress of the Covid-19 outbreak. These findings suggest that “cancer patients” are experiencing substantial adverse psychological effects as a result of the Covid-19 outbreak that are largely unrelated to their objective requirements for healthcare services. In the general population, the word “cancer” is still widely associated with the prospect of severe illness and death, despite scientific advances in our understanding of the diversity represented by this label in terms of tumor biology, clinical behaviors, and responsiveness to treatment. The sole fact that one has been diagnosed with “cancer” (regardless of the type and current status) may be largely responsible for much of the fear, anxiety, and feelings of vulnerability revealed by our survey. These results are consistent with other preliminary data (28), reporting that 71% of German cancer patients felt moderately to highly restricted in their daily life and irritated by the information distributed by the media. However, no data is yet available to compare the feelings of patients with different cancers.

Although the Italian National Institute of Health *(Istituto Superiore di Sanità)* and other health-care authorities have documented worse outcomes for Covid-19 in men and elderly individuals of either sex, the highest levels of concern over the pandemic in our cohort were expressed by women and individuals <65 years of age (Table 1). Younger age and female sex have been already emerged as risk factors for psychological distress during the Covid-19 outbreak in China. Women in particular were likely to experience anxiety, fears, depression, anger, guilt, grief and loss, post-traumatic stress, stigmatization, negative alterations in cognition or mood, and hyper-arousal (23, 29, 30).

With the advent of the Covid-19 pandemic, health-care professionals working with cancer patients find themselves faced with two seemingly unreconcilable imperatives: to provide the diagnostic and therapeutic support needed to prevent or control progression of their patients' potentially lethal neoplastic disease and to implement all strategies known to lower these patients' short-term risk of contracting a potentially lethal infection with SARS-CoV-2. It is important to keep in mind that there is relatively little evidence that currently protocols for monitoring and treating cancer are associated with significant gains in terms of survival or reduced mortality, and a “healthy” reduction in the overuse of diagnostic and therapeutic interventions might well be an unexpected benefit of the Covid-19 pandemic (31, 32). However, now is not the time to neglect our patients' fears, expectations, and need for reassurance. Fortunately, there are a wealth of digital resources available that can help us to keep in touch with and monitor our cancer patients without placing them at undue risk of infection.

To what extent these alternative forms of patient-clinician interaction will be successful in alleviating our cancer patients' fears, anxiety, and feelings of abandonment remains to be seen. It is interesting to note that, at the time our survey was conducted, when alternative forms of contact with patients had not yet been implemented, most patients in our cohort reported satisfaction with the medical support they were receiving (Table 2). This response was more frequent among patients aged 65 or older. Responses to optional questions regarding their reported attitudes frequently highlighted the value to older individuals of lessons learned during past experiences with major life challenges (including but not limited to the diagnosis of their cancer). Younger patients were more likely to express frustration and anger at shortcomings and complications arising from the Covid-19 outbreak, possibly because the fear and hardship associated with this challenge strike them as unique and unprecedented. The data collected in this survey on patient satisfaction will provide a useful baseline for evaluating the alternative forms of patient-clinician interaction and their efficacy in alleviating our cancer patients' fears during Covid-19 pandemic. In stressful times like these, listening to and learning from our patients, addressing their concerns and expectations to the best of our ability continue to be fundamental to our ability to provide high-value care.

## Data Availability Statement

All datasets presented in this study are included in the article/Supplementary Material.

## Ethics Statement

The studies involving human participants were reviewed and approved by Ethics Committee of Sapienza University of Rome. The patients/participants provided their written informed consent to participate in this study.

## Author Contributions

RF: concept, data collection, writing, and interpretation. GG: data management and interpretation and analysis and writing. VR: data collection, writing, and interpretation. RM and LG: data collection. SF: data interpretation and manuscript editing. CD: concept, data interpretation, and manuscript editing. All authors read and approved the final manuscript.

## Conflict of Interest

The authors declare that the research was conducted in the absence of any commercial or financial relationships that could be construed as a potential conflict of interest.
