# Supplementary material for: Cancer Care During COVID-19 Era: The Quality of Life of Patients With Thyroid Malignancies
Source: Front Oncol. 2020 Jun 23;10:1128. doi: 10.3389/fonc.2020.01128 (PMC7344223; doi:10.3389/fonc.2020.01128)
Supplement: Supplementary file 1 [file Data_Sheet_1.docx]

**Supplemental Figure S1**. Global Health Status/Quality of Life subscale and 5 function subscales of the EORTC QLC-C30 questionnaire: Italian general population normative data^1^ (dotted line), all patients (orange line), and Group 1 patients (grey line).

**References**

1. Nolte S, Liegl G, Petersen MA, et al. General population normative data for the EORTC QLQ-C30 health-related quality of life questionnaire based on 15,386 persons across 13 European countries, Canada and the Unites States. Eur J Cancer 2019;107:153-63.
